# Robot Voices in Daily Life: Vocal Human-Likeness and Application Context as Determinants of User Acceptance

**DOI:** 10.3389/fpsyg.2022.787499

**Published:** 2022-05-13

**Authors:** Simon Schreibelmayr, Martina Mara

**Affiliations:** LIT Robopsychology Lab, Johannes Kepler University Linz, Linz, Austria

**Keywords:** speech interface, voice assistant, human–robot interaction, synthetic voice, anthropomorphism, uncanny valley, application context, user acceptance

## Abstract

The growing popularity of speech interfaces goes hand in hand with the creation of synthetic voices that sound ever more human. Previous research has been inconclusive about whether anthropomorphic design features of machines are more likely to be associated with positive user responses or, conversely, with uncanny experiences. To avoid detrimental effects of synthetic voice design, it is therefore crucial to explore what level of human realism human interactors prefer and whether their evaluations may vary across different domains of application. In a randomized laboratory experiment, 165 participants listened to one of five female-sounding robot voices, each with a different degree of human realism. We assessed how much participants anthropomorphized the voice (by subjective human-likeness ratings, a name-giving task and an imagination task), how pleasant and how eerie they found it, and to what extent they would accept its use in various domains. Additionally, participants completed Big Five personality measures and a tolerance of ambiguity scale. Our results indicate a positive relationship between human-likeness and user acceptance, with the most realistic sounding voice scoring highest in pleasantness and lowest in eeriness. Participants were also more likely to assign real human names to the voice (e.g., “Julia” instead of “T380”) if it sounded more realistic. In terms of application context, participants overall indicated lower acceptance of the use of speech interfaces in social domains (care, companionship) than in others (e.g., information & navigation), though the most human-like voice was rated significantly more acceptable in social applications than the remaining four. While most personality factors did not prove influential, openness to experience was found to moderate the relationship between voice type and user acceptance such that individuals with higher openness scores rated the most human-like voice even more positively. Study results are discussed in the light of the presented theory and in relation to open research questions in the field of synthetic voice design.

## Introduction

Talking machines have found a place in our lives. They are supposed to assist us in a range of activities, be it performing an online search, navigating the way, or just letting us know when the spaghetti is ready. Around the world, 4.2 billion digital voice assistants, such as Amazon’s Alexa or Apple’s Siri, are already employed. By 2024, the number of digital voice assistants is predicted to reach 8.4 billion units, a number greater than the world’s human population (Juniper, 2019; Statista, 2021). Over the upcoming years, it is thus clear that ever more people will use spoken language to interact with machines—and these machines will eventually sound more and more human-like (Meinecke, 2019; Statista, 2021). Google Duplex, to mention one of the more recent innovations in the field of speech synthesis, gives us a glimpse of the future where computer voices might actually be indistinguishable from real people (Oord et al., 2016; Google Duplex, 2018). However, unlike us humans, who cannot fundamentally change the sound of our voices except for slight adaptations to the situation and interlocutor, synthetic voices are “design material” (Sutton et al., 2019) allowing for customization (Amazon, 2017; Polly, 2019; Cohn and Zellou, 2020). Depending on deliberate design decisions, computer-generated voices may thus sound more female or male, younger or old, more bored or excited—more human or mechanical.

Since virtually no new skills need to be learned for natural language communication with computers and speech interfaces are therefore considered particularly intuitive even for non-experts (e.g., Nass and Brave, 2005), synthetic voices are being used in a growing number of technological products. Besides voice assistants, these include conversational agents, customer service bots, navigation systems, social robots, vending machines, or even AI therapists (Niculescu et al., 2013; Chang et al., 2020). As voice interfaces evolve and their areas of application continue to expand, it must be ensured that the needs of users are adequately addressed. If important acceptance factors are not accounted for in their design, this may not only backfire economically, but also have negative consequences for the psychological wellbeing of users. User-centered research is therefore needed to gain a better understanding of effects of vocal human-likeness in machines and to investigate what types of synthetic voices are considered acceptable in different contexts of use.

To date, we know only little about whether realistically human-sounding computer voices would elicit particularly positive or negative user responses, and if it matters whether we think of a more social application such as a talking care robot or a more formal one such as a financial assistant. In a recent attempt to shed light on this matter, Kühne et al. (2020) found, contrary to their expectations, that participants generally liked highly human-like computer voices more than synthetically sounding ones. Against the background of the popular Uncanny Valley hypothesis (Mori, 1970) and empirical findings on visual or behavioral human-likeness in robots (Bartneck et al., 2007; Mara and Appel, 2015a,b; Appel et al., 2016; Mathur and Reichling, 2016), however, it could be assumed that a too realistic imitation of the human would lead to aversive responses.

Given the mixed perspectives in the literature, the rapidly advancing progress in the development of human-sounding synthetic voices, and the diverse purposes for which speech interfaces may be used in society, controlled user studies are required that include a range of more or less human-like voices while also considering contextual and individual differences. This is where the present work comes in with fourfold objectives. Based on a lab experiment with five different voices, supposedly belonging to a service robot, it shall contribute to answering the following questions:

*(RQ1) Voice realism and anthropomorphism:*

Are machines with more realistic voices actually more anthropomorphized than machines with less realistic voices?

*(RQ2) Human-likeness and the Uncanny Valley:*

Is the degree of perceived human-likeness related to how eerie or pleasant users evaluate a given voice?

*(RQ3) Application context and acceptance of vocal human-likeness:*

Does the acceptance of vocal human-likeness depend on the assumed application context, and more specifically on whether it is a social context?

*(RQ4) User personality and acceptance of vocal human-likeness:*

Considering tolerance of ambiguity and the Big Five personality factors, do individuals differ in how positively they evaluate vocal human-likeness?

Before we describe the conducted experiment in more detail, the underlying theoretical and empirical literature is presented in the following sections. For better comprehensibility, hypotheses are laid out directly below the literature section they were derived from.

### Human-Like Voice as Anthropomorphic Cue

The human voice is the most impactful sound in our lives. It represents a very important component of interpersonal communication and it transmits highly relevant information about its creator (Kaplan et al., 1995; McGee et al., 2001). The moment we start to speak, we automatically reveal information about our biological, psychological, and social status. Research has demonstrated that characteristics, such as a person’s gender, age, affect, and their membership in social or ethnic groups, can be inferred from the voice only, even if the person was previously unknown to the judge (Giles et al., 1979; Eagly and Wood, 1982; Kohlberg et al., 1987; Krauss et al., 2002; Pinker, 2003; Tiwari and Tiwari, 2012; Smith et al., 2016).

Looking at the crucial role of human voice to exchange information and to interpret others in our social life, it is not surprising that voice emitted by a computer is considered a particularly strong anthropomorphic cue (Nass and Brave, 2005; Qiu and Benbasat, 2009; Eyssel et al., 2012; Whang and Im, 2021), along with visual cues, such as human-like embodiment or non-verbal behavior of a machine (cf. Mara and Appel, 2015a,b). Anthropomorphism describes the widespread tendency to attribute human characteristics, motivations, intentions, or emotions to non-human entities, or in short, to sense something human where there is actually nothing human (Epley et al., 2007). This can happen with things that do not use natural speech or resemble human appearance at all, such as cuddly toys or even plants. According to Theory of Anthropomorphism of Epley et al. (2007), however, readily observable human-like features increase an object’s likelihood of being anthropomorphized because they facilitate the accessibility of anthropocentric knowledge structures and thus increase the chance that such knowledge will be applied to the non-human target. This is in line with Nass and colleagues’ Computers Are Social Actors paradigm (CASA, Nass et al., 1994; Reeves and Nass, 1996; Nass and Brave, 2005), which posits that individuals mindlessly apply social heuristics from interpersonal interactions to their interactions with computers. According to the authors, perceiving a computer as social actor is particularly likely when it takes on a role that was typically fulfilled by a human (e.g., tutor, salesperson, and therapist), when it is interactive, or when it uses natural speech (Nass et al., 1994; Nass and Brave, 2005).

In support of these theories, empirical research has found, for example, that consumers perceive voice assistants as independent agents detached from the company behind them (Whang and Im, 2021), that different voices emitted by the same computer are treated as distinct social actors (Nass et al., 1994), that the use of voice in online questionnaires elicits socially desirable responses comparable to the way a real human interviewer would (Couper et al., 2001; Tourangeau et al., 2003), and that people deduce personality cues from synthetic voice (Nass and Lee, 2001). Furthermore, initial evidence suggests that it is not just the use of voice *per se* that matters, but that greater anthropomorphization occurs with more natural computer voices than with less natural ones (Eyssel et al., 2012; Ilves and Surakka, 2013; Baird et al., 2018).

Various validated self-report scales exist to measure how much human someone sees in a machine (Bartneck et al., 2009; Ho and MacDorman, 2010; Carpinella et al., 2017). Besides, a common expression of anthropomorphism in everyday life (and also a common strategy in product marketing) is giving a human name to an object (Epley et al., 2007). Name-giving and anthropomorphism have been previously associated in the scientific literature. For example, human first names have been used to experimentally manipulate the perceived human-likeness of a machine (e.g., Qiu and Benbasat, 2009; Waytz et al., 2010). Recently, Brédart (2021) studied this relation from the flip side and revealed that people with higher anthropomorphic tendencies were also more likely to call personal objects by a proper name. While we found no existing studies on the relationship between strength of anthropomorphism and name-giving with respect to synthetic speech, there is evidence that, depending on the perceived human-likeness of a computer voice, individuals also imagine the embodiment behind the voice to be more or less human (e.g., with or without human face, hair, and hands; Mara et al., 2020), which may also reflect anthropomorphism.

From the literature presented, we derive the following initial hypotheses regarding the relationship between voice realism and anthropomorphic attributions:

*H1a*: The more realistic a voice sounds, the more *human-like* it is rated.

*H1b*: The more realistic a voice sounds, the more likely participants assign a real *human name* to the talking robot in a name-giving task.

*H1c*: The more realistic a voice sounds, the more likely participants describe the talking robot to have a *human-like appearance* in an imagination task.

### User Evaluations of Human-Like Machines: Pleasant or Uncanny?

Manufacturers of tech gadgets in many cases seek to fuel user perceptions of their products as human-like. In the context of this paper, voice assistance systems that often have not only human names but also specially created backstories (West et al., 2019), are the best example of how companies assume anthropomorphism to be associated with positive customer opinions.

Consistent with this popular belief, findings from a few recent studies indeed indicate more favorable user evaluations for greater human-likeness in computer voices. Kühne et al. (2020) drew a comparison between two currently available synthetic female voices (CereVoice, IBM Watson) and a real woman’s voice. Results indicate that the real human voice was rated as most pleasant, intelligible, likable, and trustworthy. Anecdotal evidence from two other exploratory studies suggests similar patterns Baird et al. (2018) asked 25 listeners to evaluate the likability and human-likeness of 13 synthesized male voices and found likability to increase consistently with human-likeness. Based on data from 30 listeners, also Romportl (2014) reported that most though not all participants preferred a more natural female voice over an artificial sounding one. These results are also in line with two recent meta-analyses that overall show beneficial effects of—here, mostly visual—anthropomorphic design features for embodied robots and chatbots (e.g., on affect, attitudes, trust, or intention to use), although the dependence of these effects on various moderators (e.g., robot type, task type, and field of application) points to more complex relationships between human-likeness and user responses (Blut et al., 2021; Roesler et al., 2021).

The literature, however, also features a number of studies that report non-favorable user reactions to high levels of human-likeness in machines. For example, in several experiments from the field of human-robot interaction it was found that people prefer more machine-like robot appearances over more human-like ones (Bartneck et al., 2007; Broadbent et al., 2011; Mara and Appel, 2015a,b; Mathur and Reichling, 2016; Vlachos et al., 2016; Jia et al., 2021). Works that suggest negative effects of anthropomorphic designs typically refer to the Uncanny Valley hypothesis (Mori, 1970; Mori et al., 2012), which proposes a non-linear relationship between the human-likeness of an artificial character and the valence it evokes in its observers. According to Mori’s hypothesis, in a generally low range of human-likeness, pleasantness grows with increasing realism. At a point of rather high human-likeness, however, the effect reverses and the artificial entity is perceived as eerie or threatening. Only when the entity’s degree of realism reaches near-perfection or perfection will pleasantness go up again, since no distinction can be made any longer between artificial and human (Mori et al., 2012; Mara et al., 2022). Various perceptual and cognitive mechanisms have been suggested to underlie uncanny experiences (cf. Diel and MacDorman, 2021). These include categorical uncertainty or prediction difficulties if features of a given entity seem to belong to different conceptual categories (e.g., a mechanoid robot head with a human-like voice, Mitchell et al., 2011; Meah and Moore, 2014).

In summary, given some recent empirical findings on synthetic speech, it could be assumed that voices that are perceived as more human-like are also perceived as more pleasant and less eerie (Romportl, 2014; Baird et al., 2018; Kühne et al., 2020). Against the background of the Uncanny Valley phenomenon, however, expectations would go in a different direction: On the one hand, it could be assumed that highly realistically sounding voices are evaluated as eerier and less pleasant than either a perfect imitation of the human voice or mechanically sounding voices. This would depict the curvilinear relationship between human-likeness and elicited valence as originally predicted by Mori (1970). On the other hand, if we refer to conflicting cues and categorical uncertainty as potential mechanisms behind uncanny experiences (cf. Burleigh et al., 2013; Diel and MacDorman, 2021), a mismatch between the sound of a voice (e.g., highly human-like) and available information about the speaker (e.g., “It is a robot”) could also be assumed to trigger eeriness. Since we consistently introduce each of the five voices in our study as a “robot voice,” following this idea, the real human voice might be perceived as the greatest mismatch and therefore possibly evokes greatest eeriness. Overall, given the various plausible assumptions that could be deduced from the theoretical and empirical literature, we remain with non-directional hypotheses on the relationship between voice realism, pleasantness, and eeriness at this point:

*H2a*: *Eeriness* evaluations differ between the voices and their human-likeness ratings.

*H2b*: *Pleasantness* evaluations differ between the voices and their human-likeness ratings.

### Acceptance and Application Context

Computer voices are supposed to find use in a wide variety of applications, from care or companion robots (Bendel, 2022) to AI-based financial assistants (Kaur et al., 2020). While there is hardly any research on the contextual acceptance of voice interfaces to date, recent meta-analyses from the broader field of human–robot interaction suggest that user acceptance is unlikely to be independent of the application area and the tasks for which a robot is to be used (Blut et al., 2021; Roesler et al., 2021). For example, Ullman et al. (2021) show in a series of studies that robots are consistently regarded as less trustworthy in social application contexts than in non-social ones. This is in line with an experiment, which saw the robot iCub being trusted more for functional tasks, such as image analysis than for social tasks (Gaudiello et al., 2016). Transnational surveys from Europe also indicate that many people are generally more positive about the use of robots in areas, such as space exploration or manufacturing than in areas that typically require social–communicative skills and empathy, with only 3–4% of Europeans welcoming a priority use of robots for the care of children or the elderly (Eurobarometer, 2012).

Since different application areas raise different expectations about what a machine must be able to do, it seems reasonable to assume that the degree of human-likeness considered appropriate and acceptable by users is also context-dependent. A few empirical studies have so far addressed potential interaction effects of anthropomorphism and application context. In Roesler and colleagues’ recent experiment (Roesler et al., 2022), participants had to choose one out of various robot pictures that differed in visual human-likeness based on different context descriptions. A lower degree of human-likeness was found to be preferred for industrial application and a higher degree of human-likeness for social application, while there were no clear preferences in the service domain. This is consistent with a previous study (Goetz et al., 2003), which also observed a preference for human-like robots for social tasks, but machine-like robots for investigative tasks. Oyedele et al. (2007) found tentative evidence for an interaction effect in that more human-like robots were assessed more positively in an imagined household context, while the degree of human-likeness was irrelevant for acceptance in other contexts. In contrast, results by Jung and Cho (2018) indicate no interaction as images of highly human-like robots were rated more negatively than mechanoid robots across several contexts.

Taken together, empirical findings seem to suggest that while overall acceptance for the use of robots in social application domains is lower than for non-social domains, acceptance within social applications increases with the degree to which a machine is perceived human-like. Following definitions from Social Robotics, for the purpose of this study, social applications are defined as ones in which machines act as “social partners” (Mejia and Kajikawa, 2017), engage in meaningful two-way interactions, build emotional resonance, understand human states, and respond to them according to social rules (Duffy, 2003; De Graaf et al., 2015). This was described to be the case with robots meant to provide caregiving or companionship, among others (Mejia and Kajikawa, 2017).

With respect to context-dependent differences in the acceptance of computer voices, we derive the following hypotheses from the literature:

*H3a*: Independent from voice type, *acceptance* for the use of voice interfaces is lower for social applications (care, companionship) than for non-social applications (business & finance, information & navigation).

*H3b*: The more realistic a voice sounds and the more human-like it is perceived, the more likely it is to be *accepted* for use in *social application* areas (care, companionship).

### Acceptance and User Personality

Taking personality psychological approaches into account, it can be assumed that the evaluation and acceptance (or rejection) of anthropomorphic machines is not only determined by design parameters of the machine itself and its application area, but also by user-specific factors. Two of the personality traits of the famous five-factor model (FFM or “Big Five,” Digman, 1990; John et al., 1991), namely, openness to experience and neuroticism, have been associated with the acceptance of new technologies in many studies.

Openness to experience, that is, a person’s tendency to prefer novelty over routine and to have a broad rather than a narrow range of interests, has been found to correlate, among others, with more positive attitudes toward robots (Morsunbul, 2019), acceptance of robots (Esterwood et al., 2021), acceptance of autonomous vehicles (Gambino and Sundar, 2019; Zhang et al., 2020), and with personal innovativeness in IT (Nov and Ye, 2008). In a study on a new teleworking software (Devaraj et al., 2008), openness turned out to be the only of the “Big Five” personality factors that had a direct impact on intentions to use beyond the two core predictors (usefulness, ease of use) of the widely used Technology Acceptance Model (TAM, Davis, 1989). Furthermore, people with higher openness scores were found to be less prone to technophobia (Anthony et al., 2000; Maricutoiu, 2014).

In contrast, individuals with higher neuroticism scores, that is, those who are more likely to experience emotional instability, negativity, anxiety, and irritation, showed less eagerness to adopt new technologies (e.g., Charness et al., 2018; Zhang et al., 2020) and were found to suffer more often from technophobia (Maricutoiu, 2014). Persons who scored higher in neuroticism also experienced highly human-like robots as eerier and less warm in a study (MacDorman and Entezari, 2015), which could be interpreted as a greater uncanny valley sensitivity.

Apart from the “Big Five,” initial empirical evidence indicates that persons who generally respond negatively to ambiguous stimuli or who are sensitive to a lack of structure describe highly human-like machines as eerier than others (Lischetzke et al., 2017). If a categorization process is hindered, for example due to machine characteristics that are close to categorical boundaries or due to conflicting cues (a robot as per information, but with a very natural voice), it could thus be assumed that people who score low on tolerance of ambiguity may experience discomfort or even uncanniness (cf. Bochner, 1965; Norton, 1975; Freeston et al., 1994; Furnham and Ribchester, 1995; Robinson et al., 2003; Robinson, 2004; Oshio, 2009; MacDorman and Entezari, 2015).

Based on the literature presented, we consider individual differences to play a role in user responses to human-like computer voices. Following findings from technology acceptance studies and the Uncanny Valley literature, we assume neuroticism and low tolerance of ambiguity to add to higher eeriness ratings of human-like voices, whereas greater openness to experience should add to greater acceptance for applying human-like computer voices, as reflected by the following hypotheses:

*H4a*: The relationship between perceived *human-likeness* and *eeriness* of a voice is moderated by participants’ *tolerance of ambiguity*.

*H4b*: The relationship between perceived *human-likeness* and *eeriness* of a voice is moderated by participants’ *neuroticism*.

*H4c*: Differences in user *acceptance* between the voices are moderated by participants’ *openness to experience.*

## Materials and Methods

To test our assumptions, we compared user responses to speech recordings of a total of five female-sounding voices supposed to belong to a (not visible) service robot in a randomized controlled lab experiment with constant listening conditions. In the following, we give a detailed description of the voice stimuli created for this study, the characteristics of our sample, the study procedure, and the measures used.

### Voice Stimuli

Recordings of five different voices (*human*, *synthetic I*, *synthetic II*, *metallic*, *comic*) were created as auditory stimuli. All speech samples were in German. Duration, speech content, and voice gender (female) were held constant to control for potential confounding effects. The total length of each recording was 2 min and 20 s and consisted of 306 words. The speech content represented an introduction of the history and technical functionality of robots. It was written with the intent (i) to be thematically apt but relatively neutral, (ii) not to bias the participants’ acceptance of specific robot application areas, and (iii) not to encourage anthropomorphic inferences which may systematically impact the perception of certain voice types in different ways than others (Fink et al., 2012).

In order to cover a wide range of varying vocal realism across our stimuli, recordings of a real person, professional synthetic voices as well as less realistic sounding modifications of synthetic voices were included (see Table 1). Subsequently, an overview of the five experimental voices is given.

**Table 1:** Description of the five experimental robot voices.

	Voice name	Speech engine	Modification
Real human	Human	(Pro speaker)	Breath sounds filtered
High human-likeness	Synthetic I	Amazon Polly (German)	Original version
	Synthetic II	Microsoft Hedda (German)	Original version
Low human-likeness	Metallic	Amazon Polly	Metallic effect, Echo (10%)
	Comic	Amazon Polly	Pitch shift (1.35)

#### Human

This speech sample was recorded by a professional voice-trained speaker in a quiet room using the recording software “Logic” and a large-diaphragm condenser microphone with a cardioid characteristic called “Rode NT-1 A.” As the participants were supposed to believe that this real human voice was also artificially generated, noises like exhaling and inhaling between the words were removed using the software “Adobe Audition” (Adobe Audition, 2019). This ensured that the voice sounded highly realistic yet not perfectly natural.

#### Synthetic I

In this condition, the high-quality synthetic voice “Vicki” from Amazon Polly’s text-to-speech portfolio (Polly, 2019) was used. Amazon described “Vicki” as a “voice of a similar fluency and naturalness as the German voice of Alexa” (Amazon, 2017).

#### Synthetic II

The voice “Hedda” represents an older text-to-speech system available on the Microsoft Speech Platform (Hedda, 2019). In comparison with synthetic I, this voice is more easily classified as artificial because of typically synthetic accentuations.

#### Metallic

Aiming for reduced vocal realism, here the original voice *synthetic I* was manipulated by means of a metallic echo effect (find details in Appendix A).

#### Comic

For this condition, the pitch of the original voice *synthetic I* was raised with the help of the software Voxal (2019) so that the voice sounded higher and more like a cartoon character (find details in Appendix A).

All recordings were cleaned with a manually created noise-removal filter using the software “Audacity” and adjusted to the same volume by normalizing the amplitude using the extension “dpMeter4” by “Audiveris” (Audacity, 2019; Audiveris, 2019; find details in Appendix A).

### Sample Size Justification and Participants

The sample size required for the present between-subject experiment was calculated by a power analysis using G*Power (Cohen, 1992a; Faul et al., 2007). For the calculation, a medium effect size of *f* = 0.30 was assumed and *α* error probability was set to 0.05. In order to achieve a power (1 − *β*) of 85%, the analysis resulted in a recommended sample size of at least *N* = 154 to run an ANOVA. A total of 165 German-speaking individuals took part in our lab experiment. The participants were recruited at the campus of the Johannes Kepler University in Linz, Austria and through a snowball approach.^1^ Data of two participants had to be excluded, because they reported not having responded conscientiously to all questions. Thus, the final sample consisted of 163 individuals (99 women, 64 men, no person of another or unknown gender identity), aged between 16 and 74 years (*M* = 26.39, SD = 9.64). Most of them were students (64.4%). 21.5% of participants stated they currently used a voice assistance system, such as Siri or Alexa, and 20.9% had personal experience with a robot at their home (e.g., lawn mower robot and vacuum cleaner robot). Their mean self-reported technology affinity (measured with a 5-point scale from 1 = low to 5 = high) was *M* = 3.64, SD = 1.21, overall indicating a slightly above-average interest in technology in our sample.

### Procedure

After arriving at the university’s computer lab, participants received a short introduction by the experimenter, signed a consent form, and took a seat at one of the computers. They put on high-quality over-ear headphones (Beyerdynamic DT990 Pro) and started the experiment by clicking on the computer screen. At the same time, each person was automatically assigned to one of the five voice conditions (*N*_Human_ = 34, *N*_Synthetic I_ = 34, *N*_Synthetic II_ = 33, *N*_Metallic_ = 31, *N*_Comic_ = 31). The experiment began by asking participants to provide demographic information (including age, gender, and level of education) and to fill in personality questionnaires (including Big Five traits and tolerance of ambiguity). Next, they were told that they would now hear the first part of a voice recording of a new service robot, in which they would learn about the history and technical features of robots. This initial voice recording was 1 min 20 s long. No visual stimuli were presented while participants listened to one of the voices. After the first part of the recording, participants were asked to evaluate how pleasant, human-like and eerie they found the robot voice. Subsequently, the second half of the stimulus recording with a length of 1 min was played to them, again with the same voice variant as before. In the last part of the experiment, participants rated the degree of realism of the voice and indicated how much they would accept its use in different areas of application. In addition, participants were asked to physically envision the robot they had listened to, freely describe its appearance with a few keywords, and write down an appropriate name for it. Finally, some check items were queried (e.g., answered conscientiously and quality of headphones). The entire study was conducted by use of the software Questback (2018). The experiment took about 25 min per person. Participants were fully debriefed about the research background at the end of the experimental session. No financial compensation was provided for study participation.

### Measures

#### Dependent Variables

We examined anthropomorphic attributions, eeriness, pleasantness, and acceptance as our dependent variables. The variable perceived realism was used as manipulation check (on a 9-point Likert scale).

##### Anthropomorphic Attributions

The perceived *human-likeness* of the speaking robot was assessed with five items on a five-point semantic differential scale (e.g., 1 = *synthetic*, 5 = *real*; 1 = *mechanical*, 5 = *organic*, adapted from Ho and MacDorman, 2010), which yielded an excellent reliability with Cronbach’s *α* = 0.916.

###### Assigned Name

In an open text box, participants provided a name for the robot that they felt was fitting to the robot they had listened to.

###### Imagined Embodiment

In a second open text box, participants described how they imagined the physical appearance of the robot they had listened to.

##### Eeriness and Pleasantness

*Eeriness* was measured with three items on a five-point semantic differential scale (e.g., 1 = *scary*, 5 = *comforting*, as example of an inverse coded item, adapted from Ho and MacDorman, 2010, Cronbach’s *α* = 0.765). The German items differed slightly from the English original items in favor of better comprehensibility (see Table 2, Appendix B).

*Pleasantness* was assessed by use of a single-item measure (“How pleasant did you find the voice?,” ranging from 1 = *not at all* to 5 = *very much*).

##### Acceptance

*Context-specific acceptance* was measured with the help of one item for each application context (“How much would you agree with the use of the robot you listened to in the following areas?,”—Care,—Companionship,—Information & navigation,—Business & finance;—Entertainment,—Customer service, each ranging from 1 = *not at all* to 5 = *very much*).

With this selection of listed application contexts, we attempted to cover domains (a) that have also been included in previous studies, and (b) in which voice-enabled robots or AI systems are already in use today or are expected to be increasingly used in the upcoming years (e.g., Wada et al., 2003; Wada and Shibata, 2006; Eurobarometer, 2012; Aaltonen et al., 2017; Pérula-Martínez et al., 2017; Lopatovska et al., 2019). Following our definition in chapter 1.3, the domains “care” and “companionship” were classified as social applications, while “business & finance” and “information & navigation,” where machines are usually not required to build emotional resonance or act as “social partners,” were classified as non-social applications in the context of our paper. “Entertainment” and “customer service” were included for exploratory purposes.

To compare the *cross-context acceptance* between the voices, a mean score for each voice was built by averaging the acceptance scores across all contexts.

For the *context-specific acceptance index* (including all voices), a score was created by averaging across all voices to one acceptance score for each context.

#### Moderator Variables

##### Big Five Personality Dimensions

To assess personality factors, we used a 15-item short-scale from the Socio-Economic Panel (SOEP; see Schupp and Gerlitz, 2014), based on the Big Five Inventory by John et al. (1991) and Costa and McCrae (1985). Each personality dimension is determined by three items in this scale. Internal consistencies were moderate to good (*Openness to experience:* Cronbach’s *α* = 0.73, *Conscientiousness:* Cronbach’s *α* = 0.64, *Extraversion:* Cronbach’s *α* = 0.80, *Agreeableness:* Cronbach’s *α* = 0.59, *Neuroticism:* Cronbach’s *α* = 0.70). While we had formulated hypotheses regarding the role of *openness to experience* and *neuroticism*, the other Big Five variables were included for exploratory purposes.

##### Tolerance of Ambiguity

To measure the participants’ tolerance of ambiguity we used 10 items assembled through a factor analysis by Radant and Dalbert (2003). The selection of the items is based on the 16-item short-scale developed by Schlink and Walther (2007). The scale showed a good internal consistency (Cronbach’s *α* = 0.78).

#### Manipulation Check

*Realism* was used as a manipulation check and assessed by use of a single-item measure (“How realistic does the voice of the robot sound in your opinion?,” ranging from 1 = *not at all realistic* to 9 = *very realistic*).

## Results

Before testing our hypotheses, we examined if prerequisites of parametric analyses (normal distribution, homoscedasticity of the variances) were met by our data. As this was not the case for several variables, we decided to apply non-parametric test procedures (Kruskal–Wallis tests, Spearman’s rank correlation). Significant differences in the *realism* ratings of the five voices indicate that our experimental manipulation worked [*H*(4) = 56.491, *p* < 0.001]. The real human voice was rated most realistic, the professional synthetic voices Synthetic I (by Amazon) and Synthetic II (by Microsoft) were ranked middle, and the modified synthetic voices were rated least realistic.

### Voice Realism and Anthropomorphism

We hypothesized that the five voices would be anthropomorphized to varying degrees. Along with increasing levels of voice realism, participants were expected to more likely rate a voice as human-like (*H1a*), give it a real human name (*H1b*), and imagine the (invisible) speaking robot to have a human-like physical appearance (*H1c*).

**Figure 1:**
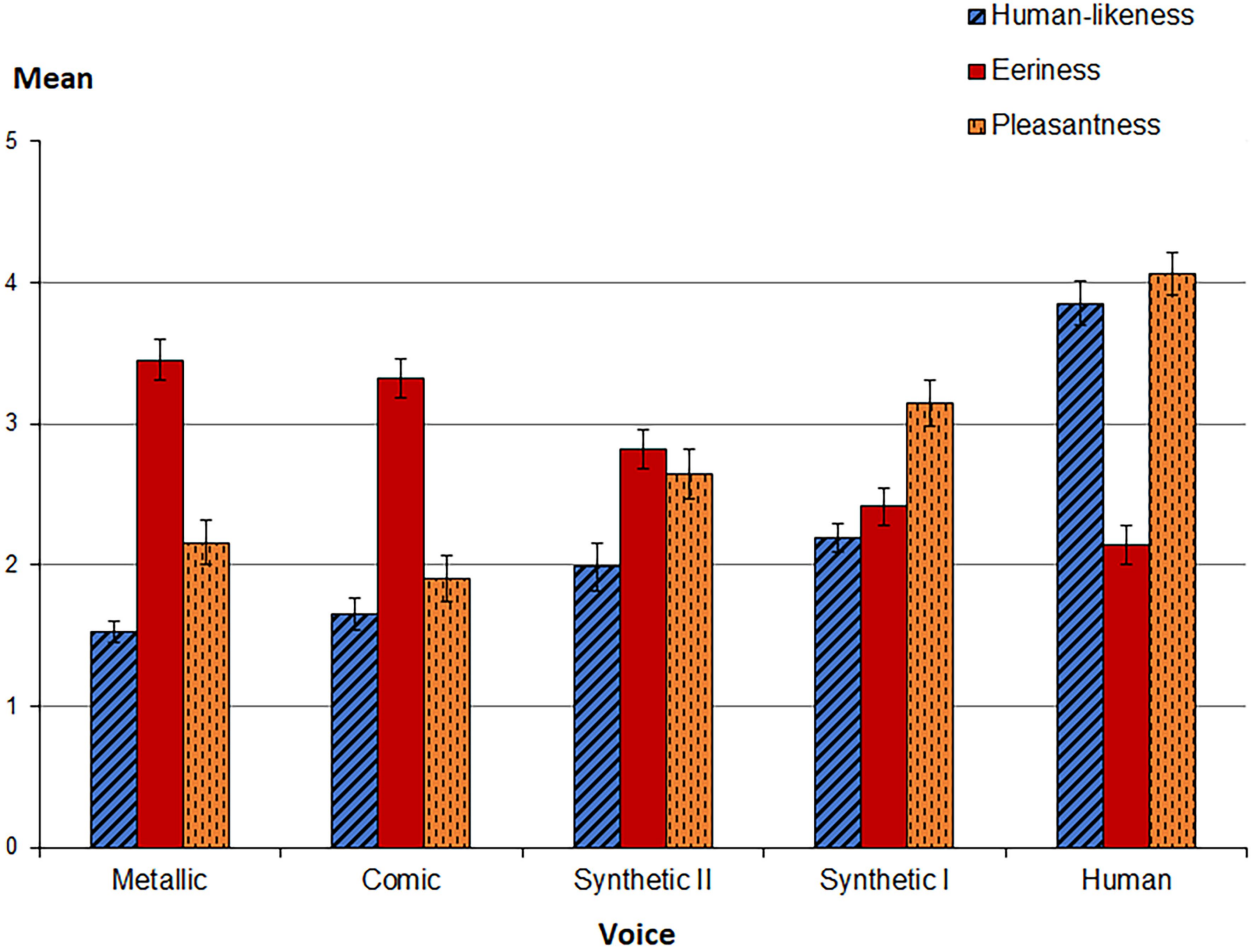
The bar chart shows the mean values of the variables *Human-likeness*, *Eeriness,* and *Pleasantness* depending on the heard voice. The five voices are arranged from left to right in an increasing degree of Human-likeness.

In terms of human-likeness ratings, significant group differences between the five voices were found [*human-likeness: H*(4) = 77.968, *p* < 0.001; see Table 2], whereby the voice *Human* is distinct from all other voices in perceived human-likeness. The highest effect size (Cohen, 1992b) is *r* = 0.96 and corresponds to a strong effect describing the difference in human-likeness between the voice *Human* (*M* = 3.85, SD = 0.93) vs. *Metallic* (*M =* 1.52, SD = 0.42). Find all pairwise group comparisons in Table 4 in Appendix C. In Figure 1, voices are ranked in the order of their perceived human-likeness.

**Table 2:** Means and standard deviations of the ratings of the five voices.

Human-likeness^*^			Eeriness^*^			Pleasantness^**^		
	Mean	SD		Mean	SD		Mean	SD
All voices	2.27	1.13	All voices	2.81	0.93	All voices	2.81	1.20
Human	3.85	0.93	Human	2.14	0.80	Human	4.06	0.89
Synthetic I	2.19	0.60	Synthetic I	2.41	0.80	Synthetic I	3.15	0.96
Synthetic II	1.99	0.97	Synthetic II	2.82	0.79	Synthetic II	2.64	1.03
Comic	1.65	0.65	Comic	3.32	0.75	Comic	1.90	0.91
Metallic	1.52	0.42	Metallic	3.45	0.79	Metallic	2.16	0.87

*N*_All_ = 163, *N*_Human_ = 34, *N*_Synthetic I_ = 34, *N*_Synthetic II_ = 33, *N*_Metallic_ = 31, *N*_Comic_ = 31.

* Rated on a five-point semantic differential scale.

** Rated on a five-point Likert scale from 1 (very unpleasant) to 5 (very pleasant).

For the analysis of assigned names, the collected names were manually classified into five categories, which we created *post-hoc* on the basis of a first check of participant responses (1 = “*female real name*,” 2 = “*male real name*,” 3 = “*existent voice assistant*,” 4 = “*fictional character*,” 5 = “*mechanical*,” *N* = 158; 5 missing). Two independent raters assigned each name to one of the classes. If they did not agree (in less than 5% of the cases), a collaborative decision was made.

**Figure 2:**
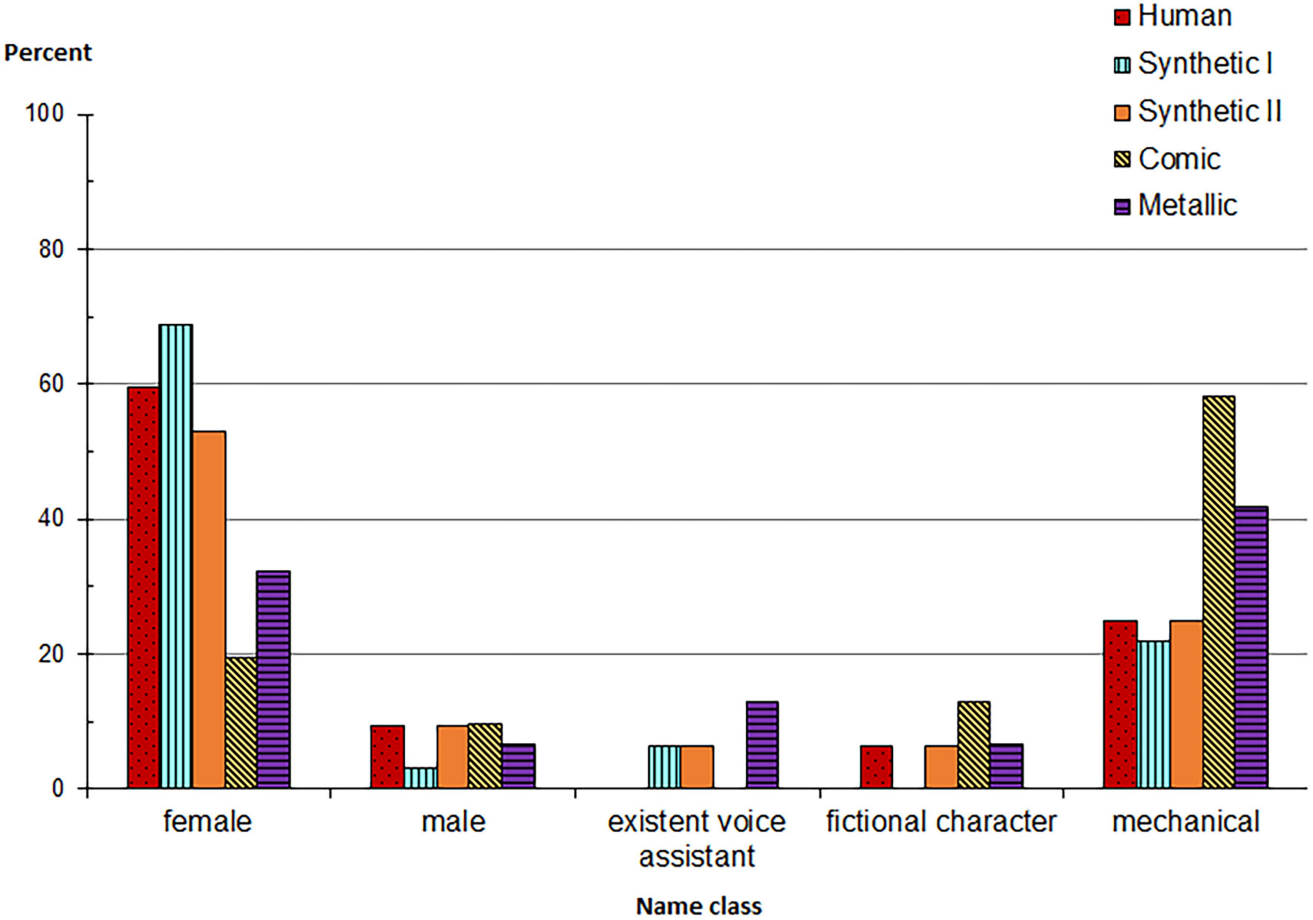
The bar chart shows the absolute values as percentage of invented names depending on the heard voice. The names were assigned to one of the five name classes.

A chi-square goodness-of-fit test revealed significant overall differences in the distribution of name classes, *Χ*^2^(4) = 117.316, *p* < 0.001. As can be seen in Figure 2 and Table 5 (see Appendix C), nearly half (45.4%) of the names that participants came up with were real female first names (e.g., “Barbara” and “Julia”), whereas about a third (33.1%) were mechanical names (e.g., “T380” and “R-74”), 7.4% were real male first names (e.g., “Robert” and “Antonius”), 6.1% fictional character names (e.g., “C3PO” and “iRobot”), and 4.9% existing speech assistants’ names (e.g., “Siri” and “Cortana”).

To test *H1b*, a chi-square test including Monte Carlo Simulation (because of insufficient cell numbers <5; Hope, 1968; Sprent, 2007) was used. As expected, significant differences were found in the distribution of chosen names between the voices, *Χ*^2^(16) = 32.360, *p* = 0.007, with the highest percentage of real human names (female/male first names) assigned to the voices Human and Synthetic I, whereas the lowest percentage of real human names was found for the voice Comic.

To test *H1c*, four independent evaluators rated the verbal descriptions of the robot’s imagined physical embodiments *post-hoc* by means of a five-point Likert scale ranging from 1 = *very mechanical embodiment* to 5 = *very human-like embodiment*. A moderate inter-rater agreement was given (Fleiss’ kappa *κ* = 0.47; Landis and Koch, 1977). After there were a couple of missing values in the embodiment descriptions, for the following group comparisons, the voices Human and Synthetic I were combined into a high vocal realism group, whereas the remaining voices Synthetic II, Comic, and Metallic were combined into a low vocal realism group. In line with our assumptions, a non-parametric Mann–Whitney *U*-test showed significant differences, indicating that the robot appearances were described as significantly more human-like after listening to one of the high vocal realism voices (Mdn = 3.5) than after listening to one of the low vocal realism voices (Mdn = 2.5), *U* = 2006.50, Z = −2.99, *p* = 0.003. Descriptions of robot appearances in the high vocal realism group included “Modelled after a female; friendly facial features and human-like behavior; blinking, head movements, female terminator?” or “female, white/light skin, blue eyes, young, cold.” Exemplary descriptions from the low vocal realism group included “Metal and plastic case, screen with text, nothing human” or “a round white disc (…); simple modern design, smooth surface.”

### Human-Likeness and the Uncanny Valley

Next, we examined our assumptions regarding the relationship between vocal human-likeness and pleasantness as well as eeriness evaluations. Our non-directional hypotheses inferred that there would be significant group differences between the voices in both their eeriness scores (*H2a*) and their pleasantness scores (*H2b*).

As expected, significant group differences between the five voices were found both for eeriness [*H*(4) = 48.468, *p* < 0.001] and for pleasantness [*H*(4) = 65.432, *p* < 0.001; See Figure 1, Table 2]. As shown in Table 6 (see Appendix C), across all voices, zero-order correlations indicate that human-likeness is negatively associated with the *eeriness* of a voice, *r_s_*(161) = −0.565, *p* < 0.01, but strongly positively associated with *pleasantness*, *r_s_*(161) = 0.699, *p* < 0.01. The real human voice was perceived as most human-like, but least eerie. *Pleasantness* and *eeriness* show a strong negative correlation, *r_s_*(161) = −0.666, *p* < 0.01. Find all significant correlations across voices as well as for each voice separately in Table 6 (see Appendix C).

After performing the Kruskal–Wallis tests, pairwise *post-hoc* comparisons were carried out for further analyses (all *p*s Dunn–Bonferroni adjusted). As shown in Table 4 (Appendix C), 5 of 10 pairwise comparisons indicate significant differences in perceived eeriness and 6 of 10 in perceived pleasantness. The greatest effect for *eeriness* with *r* = 0.70 appears in the difference between the voices *Human* vs. *Metallic*. For *pleasantness*, the greatest effect of *r* = 0.89 was found for the difference between the voices *Human* vs. *Comic*.

### Application Context and Acceptance of Vocal Human-Likeness

Regarding context-specific effects, we had hypothesized that, independent from the voice condition, acceptance for the application of a talking robot should be lower for social domains (care, companionship) than for non-social domains (business & finance, information & navigation; *H3a*), whereas with increasing realism and perceived human-likeness of a voice, its acceptance for social applications should increase (*H3b*).

A context-specific mean acceptance index was built by including values of all voice conditions. A Kruskal–Wallis test indicated a significant main effect of application context on user acceptance, *H*(5) = 309.599, *p* < 0.001. In line with *H3a*, this suggests that, independent from the type of voice, application of the talking robot was regarded most acceptable for the less social contexts of “Information & navigation” (*M* = 4.07, SD = 1.14), “Business & finance” (*M* = 3.46, SD = 1.27), “Entertainment” (*M* = 3.10, SD = 1.35), and “Customer service” (*M* = 2.84, SD = 1.30), while study participants had considerably more reservations about its use in the highly social areas “Care” (*M* = 1.98, SD = 1.13) and “Companionship” (*M* = 1.68, SD = 1.06).

**Figure 3:**
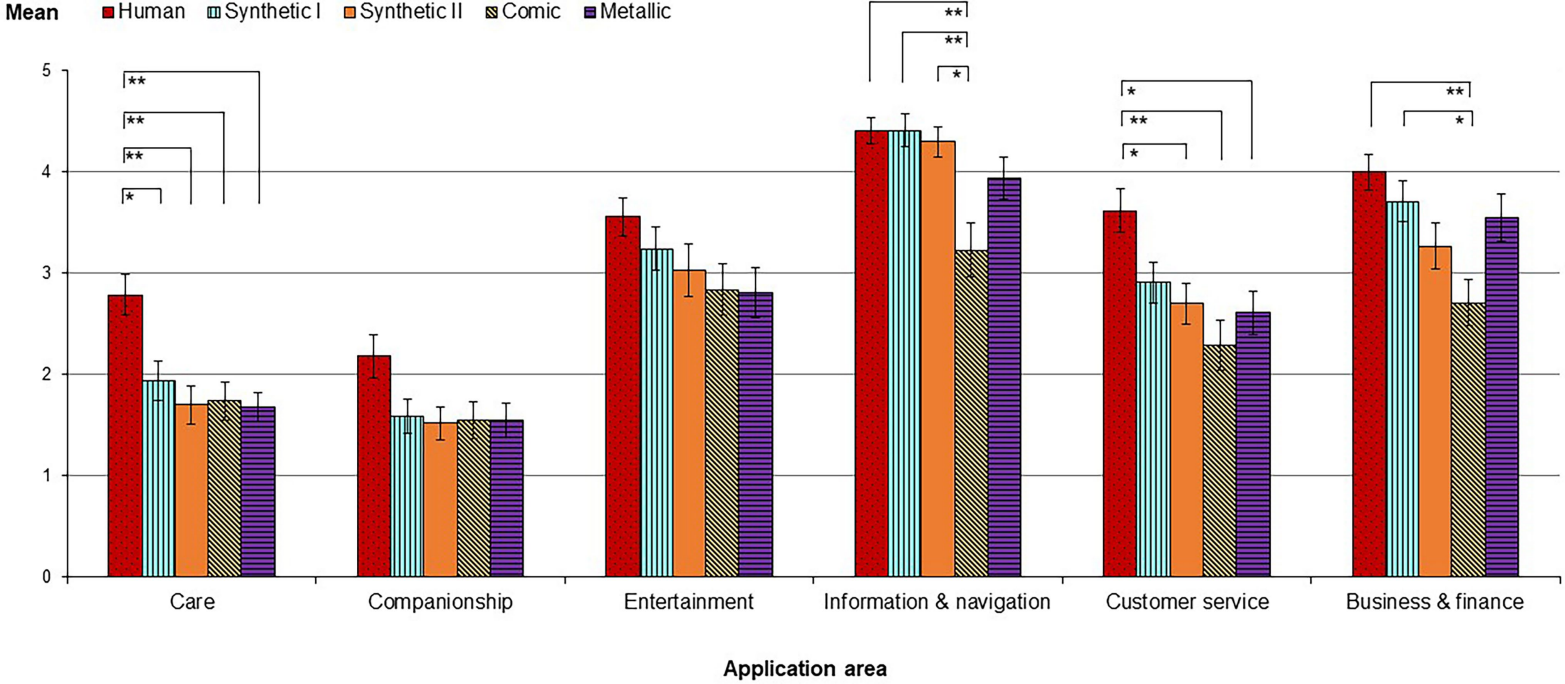
The bar chart shows the mean values of *acceptance* of the five different voices depending on the respective context. A Kruskal–Wallis test was used for pairwise group comparisons (^**^p < 0.01; ^*^p < 0.05).

Significant differences in user acceptance between the voices could be observed for five out of six contexts (Figure 3, Table 7 in Appendix C). A positive correlation between *human-likeness* of the voices and the *context-specific acceptance* was found within all application contexts. The more human-like a voice was perceived, the higher was the acceptance to use the talking service robot in the respective application area. All correlations including a 95% confidence interval based on 1,000 bootstrap samples (Davison and Hinkley, 1997; Shao and Tu, 1995) lie in a range between *r_s_* = 0.223, [0.07, 0.38], in the context of “Information & navigation” to *r_s_* = 0.386, [0.24, 0.53], in the context of “Care.” Having found a positive correlation between human-likeness and user acceptance not specifically within the social domains “Care” and “Companionship” but across all application domains, we regard *H3b* as only partially supported.

### User Personality and Acceptance of Vocal Human-Likeness

Finally, we had assumed that individual differences in tolerance of ambiguity and neuroticism would change the nature of the relationship between the perceived human-likeness and eeriness of a voice (H4a, H4b) and that differences in the participants’ openness to experience would impact the relationship between perceived human-likeness and acceptance of a voice (H4c).

Using the PROCESS macro (version 3.3) for SPSS by Andrew Hayes (Hayes and Cai, 2007; Baltes-Götz, 2017; Process, 2019), we conducted moderation analyses to examine whether *tolerance of ambiguity, neuroticism* and, for exploratory purposes, the other personality variables of the *Big Five* had a significant influence on the associations between *human-likeness* and *eeriness*. No such interactions on a significance level of *α* = 0.05 were revealed (find more information on the moderation models 1–6 in Table 8; see Appendix C). Thus, our hypotheses *H4a* and *H4b* did not find support within this study.

Additionally, moderation models were calculated for the *acceptance* over all contexts (cross-context acceptance index) with *openness to experience* and, for exploratory purposes, the other personality variables as potential moderators. Since the human voice differed significantly from the computer-generated voices in its acceptance, we created a dummy variable (real Human voice vs. all other voices) for model calculation. A confidence level of 95% was set and 5,000 samples were used for bootstrapping. A heteroscedasticity consistent standard error and covariance matrix estimator was used and continuous variables were mean-centered prior to analysis.

In support of *H4c*, a moderation model with robot voice as the predictor (Human vs. all others), *openness to experience* as the moderator, and cross-context acceptance as the outcome variable was found to be significant, *F*(3, 159) = 9.63, *p* < 0.01, *R*^2^ = 0.15. A marginal significant interaction *b* = 0.35, *t*(159) = 2.01, *p* = 0.046, indicates a positive influence through higher scores in *openness to experience* on the acceptance of the voice *Human*, but no such effect for the less realistic voices. No moderation effects were found for *tolerance of ambiguity* or the other Big Five dimensions on a significance level of *α* = 0.05 (find more information on moderation models 7–12 in Table 8; see Appendix C).

Finally, to check whether participants rated vocal human-likeness differently due to different levels of prior experience, a Kruskal–Wallis test was used to measure the influence of current usage of voice assistant systems (“Are you currently using a voice assistance system at home?”) on robot voice acceptance (cross-context). No significant differences were found between those people who are using a voice assistance system, such as Alexa or Siri, and those people who are not (Table 9; see Appendix C).

## Discussion

The human voice is an essential component of interpersonal communication and a significant influence on the formation of attitudes and opinions about others (Sporer and Schwandt, 2006; Imhof, 2010). In the age of artificial intelligence, attempts are being made to mimic natural language and human voice as closely as possible through technology. Unlike synthetic speech from earlier years, which often failed to produce convincing quality (e.g., Mayer et al., 2003; Atkinson et al., 2005), contemporary computer voices sound more and more natural (Craig and Schroeder, 2017). They prompt the idea that a phone call from a bot, for example, could soon be hardly distinguishable from a real person (Oord et al., 2016; Seaborn and Urakami, 2021)—unless a different design decision is made by the creators of the voice.

User needs and differential preferences should be taken into account early on in technology design. In light of the empirical and theoretical literature presented, however, it was left unclear whether highly realistic sounding synthetic voices were more likely to be linked to positive or negative user responses. With this study, we contribute to the understanding of how different types of voices, supposedly belonging to a service robot, are anthropomorphized, evaluated as pleasant or eerie, and accepted for real-world use. Complementing existing evidence, our randomized experiment for the first time compared assessments of five synthetic voices that differed in their degree of realism while also considering potential influences of contextual (application domain) and dispositional (personality traits) factors.

### General Discussion

Consistent with the notion that synthetic voices can serve as major anthropomorphic cues and in support of our Hypotheses 1a–c, more realistic voices were more strongly anthropomorphized than less realistic sounding voices in our experiment. This was expressed not only by higher subjective human-likeness ratings but also by the fact that more realistic voices were more often given a real human name and that study participants also imagined the robot’s embodiment to look more human-like. These results are in line with earlier work that revealed object naming as a manifestation of anthropomorphism (Qiu and Benbasat, 2009; Waytz et al., 2010; Brédart, 2021) and they also point us to potential unconscious connections between associative components of auditory and visual stimuli. Further investigations into such associative linkages may be crucial in order to create artificial voices and external object appearances that match each other (Mara et al., 2020). This is underlined by previous research, in which congruent designs of conversational machines were found to contribute to effective interaction and trust (Kiesler and Goetz, 2002; Gong and Nass, 2007; Elkins and Derrick, 2013; Torre et al., 2015, 2018).

Our non-directional Hypotheses 2a–b, stating that there would be significant group differences in pleasantness and eeriness ratings between the voices, found support in such a way that more human-like voices were experienced as significantly more pleasant and less eerie than more mechanical sounding voices. This is in agreement with prior empirical studies that also observed positive effects of anthropomorphic design features (Romportl, 2014; Baird et al., 2018; Kühne et al., 2020; Roesler et al., 2021). At the same time, it seems to contradict the Uncanny Valley hypothesis (Mori, 1970) according to which we would have expected either the quite realistic yet not perfect voices Synthetic I or II receiving the highest eeriness ratings or alternatively—assuming categorical conflicts as an important mechanism behind uncanny experiences—the real human voice (given that participants were told they were listening to a robot). What needs to be noted here is that according to Mori’s popular Uncanny Valley graph, which illustrates the assumed curvilinear relationship between human-likeness of a figure and the valence of observer evaluations, a positively valenced peak (most likable, pleasant) should occur at about 70% and a negatively valenced “valley” (most eerie, uncanny) at about 85% along the human-likeness continuum. However, with a mean value of 3.8 (on a range of 1–5) in reported human-likeness perceptions, even the real human voice in our experiment was relatively far from the right end point of the human-likeness continuum, but closer to the predicted positive peak. From this perspective, by following Mori’s postulations, it is not surprising that linear rather than curvilinear relationships between perceived human-likeness and eeriness (or pleasantness) were identified from our data, since the Uncanny Valley hypothesis itself predicts a rather linear increase of positive valence in a low to medium-high range of human similarity, that is, left of the positive peak.

Based on the collected data, it is difficult to answer why the real human voice was not rated as clearly more human-like. Perhaps filtering out the breath sounds in the actor’s speech recording (see section “Voice Stimuli”) removed an essential feature of human speech, perhaps study participants tried to resolve cognitive dissonance induced by the bad fit of the voice to the label “robot” by reporting lower perceived human-likeness (cf. Festinger, 1962; Marikyan et al., 2020), or perhaps it had to do with the general tendency of study participants to avoid endpoints of response scales (cf. Douven, 2018). A recent meta-analysis on Uncanny Valley effects of embodied humanoid robots suggests that this is a limitation not only of the current work but of many studies in the growing body of related literature. So far, there seem to be hardly any empirical studies that completely cover Mori’s human-likeness spectrum or at least make it to the almost-human level with their choice of stimuli (Mara et al., 2022). Future research on Uncanny Valley effects could therefore aim to include stimuli that are closer to the right endpoint of the human-likeness continuum and possibly also pre-test their appropriateness in pilot studies.

Regarding the context-dependent acceptance of robot voices, we found support for our hypothesis H3a. Consistent with previous surveys, in which respondents were significantly more skeptical about the use of robots or AI systems in social applications than in non-social ones (Eurobarometer, 2012; Gaudiello et al., 2016; Ullman et al., 2021), a similar pattern was also reflected in our data. On average across all voices, that is, regardless of their degree of human realism, our participants were significantly more positive about the use of a conversational robot in domains, such as information & navigation or business & finance than in the social–communicative domains care and companionship. In H3b, we had assumed that within these social domains, more human-like voices would yield particularly high acceptance scores due to a perceived congruence between the nature of such voices and typically required “human” skills in this field. After a positive correlation between human-likeness and user acceptance was found not just within social domains but across all included application scenarios, this hypothesis was only partially supported. It is worth noting, however, that the largest correlation coefficient was nonetheless observed in the highly social context of caregiving. However, we cannot completely rule out that the more realistic voices might have been perceived as particularly appropriate for use in social domains, because they also sounded more female than the mechanical voices. Due to prevailing gender stereotypes in society, women are still more often associated with communal traits (e.g., friendly, caring, and gentle) than men (Eagly and Wood, 1982; Hentschel et al., 2019). If voices that sounded more like a real woman were also unconsciously attributed more communal traits in our study, this may have led to a systematic bias in context-specific acceptance scores. To be able to detect such effects, future research is encouraged to include also male-sounding or even gender-neutral synthetic voices (cf. Carpenter, 2019) as stimuli.

While the positive influence of a participant’s openness for experience on the acceptance of vocal realism was found in line with H4c, the expected moderating roles of tolerance for ambiguity (H4a) or neuroticism (H4b) in the relationship between human-likeness and perceived eeriness of a voice were not supported by our data. We should note here that both of the latter hypotheses were based on previous findings from the empirical Uncanny Valley literature (MacDorman and Entezari, 2015; Lischetzke et al., 2017), which suggested that individuals with lower tolerance for ambiguity or higher levels of neuroticism would be particularly susceptible to uncanny effects of highly human-like machines. However, with a maximum eeriness rating of 3.45 for the voice Metallic (on a 5-point scale) and much lower eeriness scores for the more realistically sounding voices, no Uncanny Valley effect could be revealed in our study, thus the foundation for the predicted interaction effects was lacking. For individuals with low ambiguity tolerance, our initial assumption was that a possibly perceived conflict between high vocal human-likeness and the simultaneous indication that the speaker is a robot might lead to more pronounced eeriness. Our experimental manipulation did not seem to induce such a conceptual conflict, however. This could be due to the fact that even the real human voice was not rated as very much human-like on average. What, conversely, could have played a role is that a few participants in the Human voice condition expressed disbelief at the end of the study that the voice they had listened could be a robot. Future studies should therefore try to generate more convincing conflicting cues or include a measure for doubt about the presented stimulus as a control variable.

### Limitations and Outlook

Beyond the topics discussed above, we note several further limitations of the current study that may at the same time provide suggestions for future research.

First, we were only able to include five stimulus voices in our experiment, which of course cannot cover the full range of existing text-to-speech systems on the market. Although no prior study has compared such a large number of different synthetic voice types, our selection still failed to cover the human-likeness spectrum of Mori’s Uncanny Valley graph (Mori, 1970) in the higher third. Hence, it might make sense to elaborate on even more realistic sounding stimuli or on finer gradations along the vocal realism continuum. Instead of features like voice pitch as used in the current study, attempts could be made to manipulate the human-likeness of a talking robot *via* other factors, such as affective content or vocal expression.

Second, we assessed participants’ acceptance for the use of the robot voice they had listened to only by means of a self-report scale, which included one item for each application scenario. Although the items were presented in random order within our study, this makes it possible that a participant’s different contextual acceptance ratings were not independent of each other. In order to focus more closely on context-specific effects and to investigate them by means of a more rigorous study design, we propose to experimentally manipulate the supposed application area of talking machines in future work. In the frame of the current experiment, given five different voices and six application contexts (5 × 6 factorial design), this would have required a too large sample size for our lab experiment to ensure sufficient statistical power. However, future studies could focus on a smaller number of voices and create stimulus texts that target different applications for each voice.

Third, we think that the methodological approach of using pre-recorded audio files as experimental stimuli deserves some attention. While we still consider them a straightforward method to keep constant all potential influences (e.g., text content and length) apart from the voice manipulation, unidirectional listening does not represent the typical use case of synthetic voices anymore. To account for the interactivity of today’s speech interfaces, it might be worth considering having participants engage in dialog with various synthetic voices or even in live interaction with embodied talking robots.

Fourth, to advance the current line of research, it would also be valuable to go beyond cross-sectional measurements and look at user evaluations over time. Especially with very lifelike synthetic voices, it seems possible that they will raise particularly high expectations about the vividness of human–machine dialogs and the natural language capabilities of the machine. How acceptable or appropriate a synthetic voice is evaluated over time might thus also depend on how much it has been able to withstand such expectations.

Fifth, all participants in our experiment were prepared that they were about to hear a speech recording of a robot. It was not our goal to create ambiguity about the nature of the speaker. This approach is in line with current ethics guidelines for trustworthy technology (High-Level Expert Group on Artificial Intelligence, 2019), which include the requirement that conversational agents should not represent themselves as human but must disclose themselves as machines when communicating with a person. Since it can be assumed that these guidelines will not always be followed in practice, it would be interesting from both a scientific and an applied perspective to see whether a subsequent disclosure—that is, a late notice that a lifelike voice you just listened to was in fact a robot speaking—would trigger more negative user reactions, such as reactance, feelings of a loss of control or uncanny experiences. Thus, even if the participants in this study were relatively welcoming of highly human-like synthetic voices, ethical considerations and psychological consequences of intransparency may still require talking machines to be designed in a way that humans can clearly identify them as such.

## Conclusion

While technology companies deploy synthetic voices that are barely distinguishable from humans, research on user responses to different grades of vocal human-likeness in machines is still sparse. By testing effects of varying degrees of realism between five robot voices, our findings indicate that robots with more realistic sounding voices are anthropomorphized more strongly, are rated as more pleasant and less eerie, and face the highest acceptance scores across various practical application scenarios. Individuals with high openness for experience were particularly positive about the most human-like voice. Irrespective of the voice type, participants were generally more skeptical of applying talking robots to social domains that, like caregiving, require typically human skills. While this study overall suggests favorable user responses to highly human-like robot voices, a human-centered design of conversational machines certainly requires further research to build on. Beyond our cross-sectional considerations, it remains unclear whether speech interfaces can meet the high user expectations, which are likely to result from lifelike synthetic voices, in the long term. Multidisciplinary research is encouraged to look beyond technical possibilities and psychological effects also at ethical issues, which human-sounding synthetic voices ultimately raise due to their deceptive capacity.

## Data Availability Statement

The raw data supporting the conclusions of this article will be made available by the authors, without undue reservation.

## Ethics Statement

Ethical review and approval was not required for the study on human participants in accordance with the local legislation and institutional requirements. Written informed consent to participate in this study was provided by the participants’ legal guardian/next of kin.

## Author Contributions

SS and MM contributed to conception and design of the study. SS organized the database and performed the statistical analysis and wrote the first draft of the manuscript. All authors contributed to manuscript revision, read, and approved the submitted version.

## Conflict of Interest

The authors declare that the research was conducted in the absence of any commercial or financial relationships that could be construed as a potential conflict of interest.

## Publisher’s Note

All claims expressed in this article are solely those of the authors and do not necessarily represent those of their affiliated organizations, or those of the publisher, the editors and the reviewers. Any product that may be evaluated in this article, or claim that may be made by its manufacturer, is not guaranteed or endorsed by the publisher.

## Footnotes

^1^Individuals who had already participated were asked to invite new study participants. A general introductory text about the study was provided to help recruiting new participants. Persons who had already participated in the experiment were sensitized to not communicate any additional information about the contents of the study to newly recruited persons.
