# Supplementary material for: Robot Voices in Daily Life: Vocal Human-Likeness and Application Context as Determinants of User Acceptance
Source: Front Psychol. 2022 May 13;13:787499. doi: 10.3389/fpsyg.2022.787499 (PMC9136288; doi:10.3389/fpsyg.2022.787499)
Supplement: Supplementary file 1 [file Table_1.pdf]

# Appendix A

## Specific Modifications of the Voices

*Metallic.* [Pitch Shifter: Pitch Shift = 1.00; Flanger: Delay = 5 ms, Modulation Frequency = 5.00 Hz, Modulation Depth = 70%, Gain = 90%; Echo: Gain = 10%, Time = 50 ms; Amplify: Gain Percentage = 64%].

*Comic.* [Pitch Shifter: Pitch Shift = 1.35; Amplify: Gain Percentage = 136%; Compressor: Threshold = -60 dB, Ratio = 1.00, Attack Time = 20 ms, Limit = 0 dB, Release Time = 500 ms; Chorus: Gain Percentage = 80%, Delay Time = 40 ms, Modulation Frequency = 0.50 Hz, Modulation Depth = 5% ; Tremolo: Frequency = 15.00 Hz, Depth = 32%].

*Extension "dpMeter4" for "Audiveris"*

[RMS Integrated=-18 dBFS, or in older versions of the extension -18 LUFS] (See also https://developers.google.com/actions/tools/audio-loudness) (Audacity, 2019; Audiveris, 2019). The value is based on the default output of the voice Hedda. Microsoft offers a REST API and code examples for Python, which we used to generate our Hedda voice sample. The documentation, as well as examples, can be found on https://docs.microsoft.com/en-us/azure/cognitive-services/speech-service/quickstart-python-text-to-speech and in the GitHub repository [https://github.com/Azure-Samples/Cognitive-Speech-TTS/tree/master/Samples-Http/Python] (Github, 2019). The output of the web service TTSMP3.com [https://ttsmp3.com/] (which is based on Amazon Web Service (AWS) Polly) is used as an additional synthetic voice source.

## Appendix B

### Audio sample I (English translation; German original version: 1min 20sec, 170 words)

„The history of robots is long. As early as in ancient times, first attempts were made to build machines and automats. In the second half of the twentieth century, robots experienced a boom, especially in industry. Now, at the beginning of the twenty-first century, robots are moving closer to humans. Their application is considered in more and more fields and they are entering various areas of work and personal life. Robots like me, which provide services for humans, are called service robots. As a service robot, I have basic technical functions with which I should be able to support people in different areas of application on a preferably universal level. This requires a combination of the latest technologies from the areas of kinematics, sensors, machine learning and artificial intelligence. In the future, my linguistic abilities will allow users to speak to me as if they were talking to another person. This will make me suitable for users without any prior technical knowledge. No special training will be required to operate me."

### Audio sample II (English translation; German original version: 1min, 136 words)

„Earlier on, I explained a few things about the development of robots and now I would like to tell you a bit more about my abilities and areas of application. I can be used stationary or move freely around the room. I learn from my interactions with people and will be able to better assess the needs of my users over time. Furthermore, I have an image recognition system and memory. This means that I can recognize a person and retrieve data from previous interactions with them. Since I am able to access specific information or space coordinates on the Internet in real time, I can usually answer questions from my users quickly and precisely. My ability to learn and to search through large amounts of data very quickly for suitable information makes me a good advisor and knowledgeable companion in a wide variety of application contexts."

**Table 2**

*Translations of the Uncanny-Valley Items*

| | **English** | **German** |
|---|---|---|
| **Human-likeness** | artificial – natural | künstlich – natürlich |
| | synthetic – real | synthetisch – echt |
| | living – inanimate | lebendig – unbelebt |
| | humanlike – inhuman | menschlich – unmenschlich |
| | mechanical – biological | mechanisch – organisch |
| **Eeriness** | eerie – reassuring | furchterregend – beruhigend |
| | predictable – thrilling | behaglich – unheimlich |
| | bland – uncanny | nicht gruselig – gruselig |

# Appendix C

**Table 4**

*Pairwise Comparisons (Human-likeness)*

| Sample I – Sample II | Test Statistic | Std. Error | Std. Test Statistic | Sig. | Adj. Sig. | Effect size r |
|---|---|---|---|---|---|---|
| Human – Synthetic I | -48.044 | 11.405 | -4.212 | .000 | .000** | .51 |
| Human – Synthetic II | -68.340 | 11.491 | -5.947 | .000 | .000** | .73 |
| Human – Metallic | -89.939 | 11.678 | -7.702 | .000 | .000** | .96 |
| Human – Comic | -83.246 | 11.678 | -7.128 | .000 | .000** | .88 |
| Synthetic I – Synthetic II | 20.295 | 11.491 | 1.766 | .077 | .774 | |
| Synthetic I – Metallic | 41.895 | 11.678 | 3.588 | .000 | .003** | .45 |
| Synthetic I – Comic | 35.202 | 11.678 | 3.014 | .003 | .026* | .37 |
| Synthetic II – Metallic | -21.600 | 11.762 | 1.836 | .066 | .663 | |
| Synthetic II – Comic | -14.906 | 11.762 | 1.267 | .205 | 1.000 | |
| Metallic – Comic | -6.694 | 11.944 | -.560 | .575 | 1.000 | |

*Pairwise Comparisons (Eeriness)*

| Sample I – Sample II | Test Statistic | Std. Error | Std. Test Statistic | Sig. | Adj. Sig. | Effect size r |
|---|---|---|---|---|---|---|
| Human – Synthetic I | 12.529 | 11.376 | 1.101 | .271 | 1.000 | |
| Human – Synthetic II | 34.049 | 11.462 | 2.971 | .003 | .030* | .36 |
| Human – Metallic | 65.690 | 11.648 | 5.640 | .000 | .000** | .70 |
| Human – Comic | 59.771 | 11.648 | 5.131 | .000 | .000** | .64 |
| Synthetic I – Synthetic II | -21.520 | 11.462 | -1.878 | .060 | .604 | |
| Synthetic I – Metallic | -53.161 | 11.648 | -4.564 | .000 | .000** | .57 |
| Synthetic I – Comic | -47.241 | 11.648 | -4.056 | .000 | .000** | .50 |
| Synthetic II – Metallic | 31.641 | 11.732 | 2.697 | .007 | .070 | |
| Synthetic II – Comic | 25.721 | 11.732 | 2.192 | .028 | .283 | |
| Metallic – Comic | 5.919 | 11.914 | .497 | .619 | 1.000 | |

*Pairwise Comparisons (Pleasantness)*

| Sample I – Sample II | Test Statistic | Std. Error | Std. Test Statistic | Sig. | Adj. Sig. | Effect size r |
|---|---|---|---|---|---|---|
| Human – Synthetic I | -32.000 | 11.102 | -2.882 | .004 | .039* | .35 |
| Human – Synthetic II | -51.952 | 11.185 | -4.645 | .000 | .000** | .57 |
| Human – Metallic | -70.637 | 11.367 | -6.214 | .000 | .000** | .77 |
| Human – Comic | -81.298 | 11.367 | -7.152 | .000 | .000** | .89 |
| Synthetic I – Synthetic II | 19.952 | 11.185 | 1.784 | .074 | .745 | |
| Synthetic I – Metallic | 38.637 | 11.367 | 3.399 | .001 | .007** | .42 |
| Synthetic I – Comic | 49.298 | 11.367 | 4.337 | .000 | .000** | .54 |
| Synthetic II – Metallic | -18.685 | 11.449 | -1.632 | .103 | 1.000 | |
| Synthetic II – Comic | -29.346 | 11.449 | -2.563 | .010 | .104 | |
| Metallic – Comic | 10.661 | 11.626 | .917 | .359 | 1.000 | |

* $p < .05$; ** $p < .01$

*Each row tests the null hypothesis that the sample I and sample II distributions are the same.*
*Asymptotic significances (2-sided tests) are displayed. Significance values have been adjusted by the Bonferroni correction for multiple tests.*

**Table 5**

*Voice * Name class Crosstabulation*

| Voice | | Name class | | | | | Total |
|---|---|---|---|---|---|---|---|
| | | **Female** | **Male** | **Existent voice assistant** | **Fictional character** | **Mechanical** | |
| Human | Count | 19 | 3 | 0 | 2 | 8 | 32 |
| | Exp. Count | 14.99 | 2.43 | 1.62 | 2.03 | 10.94 | 32 |
| | % within Voice | 59.38 | 9.38 | .00 | 6.25 | 25.00 | 100 |
| | Adj. Residual | 1.59 | .43 | -1.46 | -.02 | -1.23 | |
| Synthetic I | Count | 22 | 1 | 2 | 0 | 7 | 32 |
| | Exp. Count | 14.99 | 2.43 | 1.62 | 2.03 | 10.94 | 32 |
| | % within Voice | 68.75 | 3.13 | 6.25 | .00 | 21.88 | 100 |
| | Adj. Residual | 2.78 | -1.07 | .34 | -1.65 | -1.64 | |
| Synthetic II | Count | 17 | 3 | 2 | 2 | 8 | 32 |
| | Exp. Count | 14.99 | 2.43 | 1.62 | 2.03 | 10.94 | 32 |
| | % within Voice | 53.13 | 9.38 | 6.25 | 6.25 | 25.00 | 100 |
| | Adj. Residual | .80 | .43 | .34 | -.02 | -1.23 | |
| Comic | Count | 6 | 3 | 0 | 4 | 18 | 31 |
| | Exp. Count | 14.52 | 2.35 | 1.57 | 1.96 | 10.59 | 31 |
| | % within Voice | 19.35 | 9.68 | .00 | 12.90 | 58.06 | 100 |
| | Adj. Residual | -3.42 | .49 | -1.43 | 1.68 | 3.13 | |
| Metallic | Count | 10 | 2 | 4 | 2 | 13 | 31 |
| | Exp. Count | 14.52 | 2.35 | 1.57 | 1.96 | 10.59 | 31 |
| | % within Voice | 32.26 | 6,45 | 12.90 | 6.45 | 41.94 | 100 |
| | Adj. Residual | -1.81 | -.27 | 2,22 | .03 | 1.02 | |
| **Total** | Count | 74 | 12 | 8 | 10 | 54 | 158 |
| | Exp. Count | 74 | 12 | 8 | 10 | 54 | 158 |
| | % within Voice | 46.84 | 7.59 | 5.06 | 6.33 | 34.18 | 100 |

*15 cells (60.0%) have expected count less than 5. The minimum expected count is 1.57.*
*Based on 10000 sampled tables. The standardized statistic is -.268.*
*N = 158*

**Table 6**

*Spearman correlation matrix over all voices*

| | **Eeriness** | **Pleasantness** | **Age** | **Gender** |
|---|---|---|---|---|
| **Human-likeness** | | | | |
| (All voices) | -.565** | .699** | | |
| Only: Human | -.535** | .376* | | |
| Synthetic I | | -.407* | | |
| Synthetic II | -.516** | .569** | | |
| Comic | | .437* | -.421* | |
| Metallic | | | | |
| **Eeriness** | | | | |
| (All voices) | | -.666** | | |
| Only: Human | | -.655** | | |
| Synthetic I | | -.450** | -.369* | -.340* |
| Synthetic II | | -.496** | | |
| Comic | | | | |
| Metallic | | -.635** | | |
| **Pleasantness** | | | | |
| (All voices) | -.666** | | | |
| Only: Human | -.655** | | | |
| Synthetic I | -.450** | | | |
| Synthetic II | -.496** | | | |
| Comic | | | -.438* | |
| Metallic | -.635** | | | |

*Only significant correlations displayed. [a]Gender is a dummy variable with values: 1 = female, 2 = male, 3 = another or unknown gender identity.*

**Table 7**

*Test Statistics*[a,b]

| | Care | Companion-ship | Entertainment | Information & navigation | Customer service | Business & finance |
|---|---|---|---|---|---|---|
| Kruskal-Wallis H | 21,911 | 9,911 | 6,725 | 18,088 | 18,505 | 18,558 |
| df | 4 | 4 | 4 | 4 | 4 | 4 |
| Asymp. Sig. | ,001 | ,041 | ,151 | ,001 | ,001 | ,001 |

*a. Kruskal Wallis Test*

*b. Grouping Variable: Voice*

**Table 8**

*Moderation-models 1-6: x = human-likeness, y = eeriness*

| | | |
|---|---|---|
| **Model 1:** M = tolerance of ambiguity | F(3, 159) = 34.54, p < .01, $R^2$ = .33 | *b* = -.10, *t*(159) = -1.84, *p* = .067 |
| **Model 2:** M = agreeableness | F(3, 159) = 35.67, p < .01, $R^2$ = .33 | *b* = .09, *t*(159) = 1.23, *p* = .220 |
| **Model 3:** M = consciousness | F(3, 159) = 32.84, p < .01, $R^2$ = .32 | *b* = .00, *t*(159) = 0.04, *p* = .969 |
| **Model 4:** M = extraversion | F(3, 159) = 32.48, p < .01, $R^2$ = .32 | *b* = -.02, *t*(159) = -0.40, *p* = .690 |
| **Model 5:** M = neuroticism | F(3, 159) = 38.11, p < .01, $R^2$ = .35 | *b* = -.06, *t*(159) = -1.17, *p* = .244 |
| **Model 6:** M = openness to exp. | F(3, 159) = 35.74, p < .01, $R^2$ = .33 | *b* = -.10, *t*(159) = -1.50, *p* = .136 |

*Moderation-models 7-12: x = voice (human vs. other voices), y = acceptance*

| | | |
|---|---|---|
| **Model 7:** M = tolerance of ambiguity | F(3, 159) = 9.36, p < .01, $R^2$ = .15 | *b* = -.01, *t*(159) = -0.05, *p* = .962 |
| **Model 8:** M = agreeableness | F(3, 159) = 12.55, p < .01, $R^2$ = .14 | *b* = -.08, *t*(159) = -0.45, *p* = .653 |
| **Model 9:** M = consciousness | F(3, 159) = 8.21, p < .01, $R^2$ = .13 | *b* = -.01, *t*(159) = -0.02, *p* = .981 |
| **Model 10:** M = extraversion | F(3, 159) = 13.05, p < .01, $R^2$ = .15 | *b* = .31, *t*(159) = 1.64, *p* = .102 |
| **Model 11:** M = neuroticism | F(3, 159) = 7.83, p < .01, $R^2$ = .13 | *b* = -.05, *t*(159) = -0.25, *p* = .807 |
| **Model 12:** M = openness to exp. * | F(3, 159) = 9.63, p < .01, $R^2$ = .15 | *b* = .35, *t*(159) = 2.01, *p* = .046* |

** Significance interaction, p < .05*
*A confidence-level of 95% was set and 5000 samples were used for bootstrapping. A heteroscedasticity consistent standard error and covariance matrix estimator was used and continuous variables were mean centered prior to analysis.*

**Table 9**

*Test Statistics[a,b]*

| | Acceptance | Human-likeness | Eeriness | Pleasantness |
|---|---|---|---|---|
| Kruskal-Wallis H | 0,322 | 0,837 | 0,665 | 0,128 |
| df | 1 | 1 | 1 | 1 |
| Asymp. Sig. | ,571 | ,360 | ,415 | ,720 |

*a. Kruskal Wallis Test*
*b. Grouping Variable: Previous experience*

## REFERENCES


Audacity (2019). Available at: https://www.audacityteam.org/ (Accessed April 20, 2019).

Audiveris (2019). Tool for audio-loudness. Available at: https://developers.google.com/Actions/tools/audio-loudness (Accessed February 12, 2019).

Github (2019). Python. Available at: https://github.com/Azure-Samples/Cognitive-Speech-TTS/tree/master/Samples-Http/Python (Accessed February 02, 2019).
